# Clinical effectiveness and cost-effectiveness of two sodium fluoride varnish systems in preventing dental caries in children: a randomized clinical trial

**DOI:** 10.1016/j.jobcr.2025.11.018

**Published:** 2025-12-02

**Authors:** Mahesh R. Khairnar, P.G. Naveen Kumar, Harloveen Sabharwal, Sachin Kumar Jadhav, Sheetal Badnaware, Neha Shukla, Ananta Kusumakar, Zainab Akram, Savitha Priyadarsini S, Ridhi Ghodela

**Affiliations:** a Public Health Dentistry, Faculty of Dental Sciences, IMS, Banaras Hindu University, Varanasi, India; b Public Health Dentistry, ESIC Dental College, Gulbarga, India; c Paediatric and Preventive Dentistry, Faculty of Dental Sciences, IMS, Banaras Hindu University, Varanasi, India

**Keywords:** Cost-effectiveness, Dental caries prevention, Dental varnishes, Fluorides, Topical, Randomized controlled trial

## Abstract

**Background:**

Dental caries frequently affects first permanent molars soon after eruption due to their anatomical susceptibility. Fluoride varnish is a widely accepted preventive measure; however, different formulations vary in fluoride retention and cost. Evidence comparing both clinical outcomes and economic value of available varnishes in children remains limited.

**Aim:**

To compare the clinical effectiveness and cost-effectiveness of resin-based and alcohol-based 5 % sodium fluoride varnishes in preventing dental caries in permanent first molars of high-risk schoolchildren.

**Design:**

A double-blind, parallel-arm randomized controlled trial was conducted among 84 children (330 M) aged 6–8 years from a private school in Varanasi, India. Participants received either resin-based varnish (163 teeth) or alcohol-based varnish (167 teeth) at six-month intervals. Caries incidence was assessed at baseline, 6, 12, 18, and 24 months using ICDAS-II. A financial cost-benefit perspective was adopted, considering direct material and application costs borne by the provider and estimated restorative treatment savings for patients. Appropriate statistical tests were used for analysis (α = 0.05).

**Results:**

Caries incidence remained low in both groups after 24 months (resin-based: 7.4 %; alcohol-based: 7.8 %; p = 0.961). No significant differences were found in early or advanced lesions at any interval (p > 0.05). ITT and Per-protocol analyses revealed negligible effect sizes (Cliff's Delta ≤0.05). Cost-effectiveness analysis demonstrated that the alcohol-based varnish produced ₹62.22 savings per ₹1 spent, compared to ₹8.47 in resin-based group. The cost to save ₹100 in future restorative expenses was ₹1.61 (alcohol-based) and ₹11.80 (resin-based).

**Conclusion:**

Both varnishes were equally effective, but alcohol-based varnish was substantially more cost-effective, making it a suitable choice for large-scale preventive programs in resource-constrained settings.

## Highlights

• First RCT assessing caries-preventive efficacy of an alcohol-based 5% sodium fluoride varnish in children.
• A 24-month double-blind RCT compared resin-based and alcohol-based fluoride varnishes in 6–8-year-old children.
• Both varnish types showed comparable caries-preventive efficacy, with over 92 % caries-free survival at 2 years.
• No significant differences were found in early or advanced lesion development between the varnish groups.
• Alcohol-based varnish was over seven times more cost-effective, supporting use in large school-based programs.

## Graphical abstract

**Figure:**
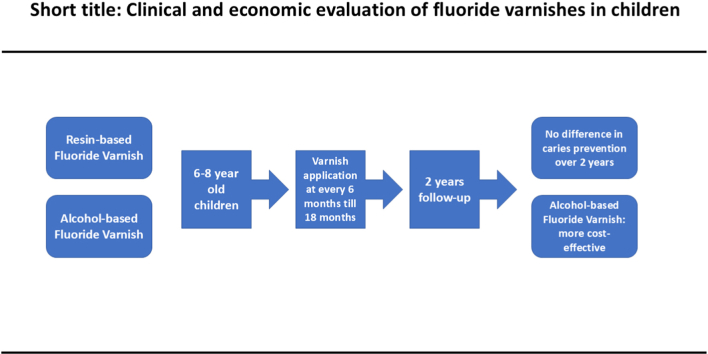

## 1 Introduction

Dental caries, a prevalent oral disease, are frequently seen in school-aged children.^1^ Despite being preventable, dental caries continue to be the most widespread chronic condition among children aged 6–11 years and adolescents aged 12–19 years.^2^ The age range of 6–14 years, characterized by mixed dentition, is ideal for primary intervention, as permanent teeth are erupting, and by evaluating oral hygiene, measures can be implemented to avert caries in the permanent dentition. Preventing dental caries in kids and teens is typically seen as a key focus for dental services and is viewed as more cost-efficient than treating it. Consequently, public health prioritizes preventive approaches aimed at school-aged children, as schools provide a structured and accessible environment to effectively deliver oral health interventions at scale.^2^^,^^3^

Fluoride varnish plays a major role in reducing enamel demineralization and enhancing remineralization, and its professional application is widely recommended for caries prevention in children.^4^, ^5^, ^6^ First permanent molars are particularly vulnerable during early eruption due to immature enamel and deep occlusal morphology, making them an ideal target for school-based preventive programs.^7^^,^^8^ Among the available varnish options, resin-based formulations are created to enhance fluoride retention but tend to be expensive and require more precise application techniques.^9^^,^^10^ In contrast, alcohol-based varnishes offer simpler handling, faster drying capability, and lower cost, making them suitable for large-scale school-based delivery, especially in resource-limited settings.^11^^,^^12^ Directly comparing these two varnish systems is therefore clinically relevant to determine whether the additional cost and application complexity of resin-based varnish translate into superior caries prevention outcomes.

Integrating cost-effectiveness with clinical outcomes is essential for informed decision-making in public oral health programs to ensure that preventive interventions are both affordable and beneficial for the population.^13^^,^^14^ Thus, this randomized clinical trial evaluated the clinical effectiveness and cost-effectiveness of resin-based versus alcohol-based 5 % sodium fluoride varnish in preventing dental caries in first permanent molars of children aged 6–8 years within a school-based program.

**Primary Objective:** To compare the clinical effectiveness of resin-based and alcohol-based 5 % sodium fluoride varnishes in preventing dental caries in newly erupted first permanent molars among school-aged children using ICDAS-II criteria over a 24-month period.

**Secondary Objective:** To evaluate the cost-effectiveness of the two fluoride varnish systems by assessing the cost-to-savings ratio and return on investment from a societal and patient perspective, based on avoided restorative treatment needs.

**Null Hypothesis:** There is no significant difference in clinical effectiveness and cost-effectiveness between resin-based and alcohol-based sodium fluoride varnishes.

## 2 Material and methods

**Study Design:** This was a parallel-arm, double-blind randomized controlled trial designed to evaluate the clinical effectiveness and cost-effectiveness of two sodium fluoride varnish formulations over 24-month period. The clinical effectiveness findings were reported in accordance with the CONSORT 2022 guidelines, and the economic evaluation followed the CHEERS 2022 checklist.

**Study setting and Participants:** This study was conducted in a private school located in Varanasi, India. Children between the ages of 6 and 8 were selected through probability sampling to find eligible participants.

**Inclusion criteria:** School children aged 6–8 years with fully erupted and caries-free first permanent molars, whose parents or legal guardians provided informed written consent, and who were classified as high caries risk according to AAPD guidelines (presence of one or more carious lesions in the primary dentition; deft >0) were included.

**Exclusion criteria:** Children with decayed or prosthetically treated first permanent molars, those affected by ulcerative gingivitis or stomatitis, and those with clinically non-visible or unerupted first permanent molars were not included.

**Ethical Considerations:** This study was performed in accordance with the Declaration of Helsinki. This study was performed in accordance with the Declaration of Helsinki. This human study was approved by Institutional Ethics Committee, Institute of Medical Sciences, Banaras Hindu University, Varanasi (Approval Number: Dean/2022/EC/3621). This trial was registered with Clinical Trial Registry – India (CTRI/2023/08/056501). Written parental informed consent and verbal assent from children were obtained prior to participation.

**Study Period:** The trial was conducted from August 2023 to August 2025, including baseline examinations and follow-ups at 6-month intervals.

**Sample Size:** The required sample size was calculated based on a Cohen's medium effect size (d = 0.5), with a Type I error (α) of 0.05 and power (1 - β) of 90 %. The initial estimation yielded a sample size of 86 teeth per group. Accounting for potential dropouts due to the extended follow-up period, the final sample was increased to 100 teeth per group.

**Examiner Calibration and Blinding:** Two experienced dental professionals underwent training in ICDAS-II scoring under a gold-standard examiner. Intra-examiner agreement was excellent (Cohen's kappa: 0.81 and 0.79) and inter-examiner agreement was strong (kappa = 0.803). The clinical evaluator applying the varnish and the outcome assessor recording ICDAS-II scores were different individuals to ensure masked assessment. Both assessors and participants remained blinded to group allocation**.**

**Randomization:** Randomization was performed using an online tool available at https://ctrandomization.cancer.gov/. Allocation concealment was performed using sequentially numbered opaque sealed envelop method wherein the principal investigator opened the envelopes with group allocation codes just before the application of the respective varnish. An investigator, who was not the part of the main study, prepared the envelopes with group allocation codes based on the randomization list received. A total of 84 participants were allocated equally (1:1 ratio) into two intervention groups, namely, Resin-based 5 % Sodium Fluoride Varnish (Profluorid Varnish; VOCO, Germany) and Alcohol-based 5 % Sodium Fluoride Varnish (Fluoritop SR; ICPA, India). Randomization was performed at the participant level, with children allocated 1:1 to receive either resin-based or alcohol-based fluoride varnish. However, the unit of analysis for clinical effectiveness was the permanent first molar, as each child contributed a maximum of four teeth for evaluation. Because teeth within the same child may exhibit intra-individual correlation, clustered data were handled by applying conservative statistical tests based on categorical frequencies at the tooth level, and both Intention-to-Treat (ITT) and Per-Protocol (PP) analyses were conducted to ensure robustness of findings.

**Intervention and follow-up:** Eighty-four children (330 first permanent molars) were randomly allocated (1:1) to receive either resin-based fluoride varnish (Profluorid Varnish, VOCO, Germany) or alcohol-based varnish (Fluoritop SR, ICPA, India). Varnish was applied at baseline, 6, 12, and 18 months. Initial clinical assessments were succeeded by evaluations at 6, 12, 18, and 24 months. All participants received standard preventive care including professional cleaning, oral hygiene instructions, and dietary advice. Children were instructed to maintain routine toothbrushing with fluoride toothpaste but no additional fluoride therapies or sealants were allowed during the study period. No pilot study preceded the clinical trial.

**Outcome measures:** The occurrence of new carious lesion in the designated first permanent molar was evaluated using the International Caries Detection and Assessment System II (ICDAS-II) at 6-month intervals over a period of 24 months, which was regarded as the main outcome.^15^ The cost-effectiveness of each intervention, assessed according to the overall cost of varnish utilized and the quantity of caries averted in every group, was regarded as the secondary outcome.

**Statistical analysis:** Data from the study were compiled in Microsoft Excel and analyzed using SPSS Version 26 with a 5 % significance level. Descriptive statistics summarized demographic data. Caries incidence was assessed using ICDAS-II criteria, where scores 0–1 were non-carious and ≥2 were carious. Due to low expected frequencies, Fisher's Exact Test compared caries occurrence between resin-based and alcohol-based varnish groups at 6, 12, 18, and 24 months. Lesions were categorized as sound (score 0), early (1–2), or advanced (3–6), and group comparisons were again made using Fisher's Exact Test. Kaplan–Meier survival analysis determined time to first carious event within 24 months, with survival curves compared via the log-rank test and Chi-square statistics. Both Intention-to-Treat (ITT) and Per-Protocol (PP) analyses were performed; ITT employed the Last Observation Carried Forward method to handle missing data. Caries score differences between groups were analyzed using the Mann–Whitney *U* test due to non-normal data distribution, and effect sizes were calculated using Cliff's Delta. Despite the consideration of multilevel modelling, the low incidence of caries events and similar exposure conditions among teeth within each participant supported the use of tooth-level analysis, as recommended in comparable fluoride varnish trials.

**Economic Evaluation:** A financial cost-benefit perspective from the provider and patient expenditure viewpoint was adopted for the economic assessment. The analysis included direct intervention costs (varnish material and application) and projected treatment savings based on avoided restorative procedures. Total varnish cost (material and application) and cost savings from prevented restorative treatment (₹2000 per tooth) were considered. A cost-to-savings ratio and the cost required to save ₹100 in future restorative expenses were calculated for each group, in alignment with CHEERS recommendations. The costing inputs used in calculations are provided as Supplementary Table S1.

## 3 Results

**Fig. 1:**
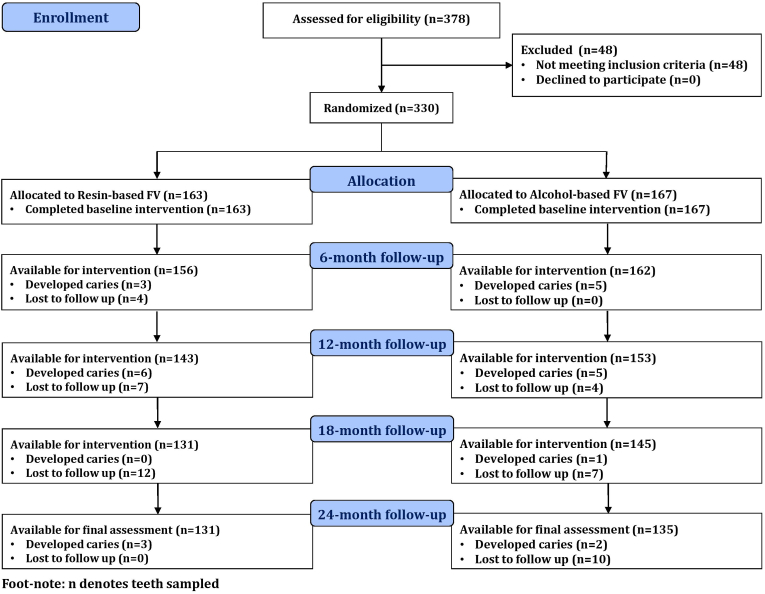
CONSORT Flow Diagram Flow diagram showing enrollment, allocation, follow-up, and analysis of participants in resin-based and alcohol-based fluoride varnish groups over the 24-month trial period.

A total of 378 teeth were assessed for eligibility, of which 48 were excluded for not meeting inclusion criteria. A flow chart illustrating the participant and tooth progression throughout the study is presented as Fig. 1, with a footnote specifying that n denotes teeth sampled. The remaining 330 teeth were randomized into two groups: resin-based FV (n = 163) and alcohol-based FV (n = 167). All randomized teeth completed the baseline intervention. Follow-up assessments were performed at 6, 12, 18, and 24 months. Attrition occurred due to teeth lost to follow-up at successive intervals, while some teeth developed caries during the observation period. At the final 24-month follow-up, 131 teeth remained available for assessment in the resin-based FV group and 135 in the alcohol-based FV group.

Table 1 shows the demographic features of participants in the fluoride varnish (FV) groups using resin-based and alcohol-based formulas. Eighty-four children took part in the study, split between the resin-based fluoride varnish group (n = 163 teeth) and the alcohol-based fluoride varnish group (n = 167 teeth). The demographic features of each group were similar regarding age and gender distribution. The average age of participants in the resin-based and alcohol-based groups was 7.48 ± 0.55 years and 7.69 ± 0.47 years, respectively. The distribution of genders was nearly identical in both groups. Table 2 examines the incidence of caries in two groups (resin-based versus alcohol-based fluoride varnish) at various time intervals through the Fisher's exact test. Throughout all follow-up periods (6, 12, 18, and 24 months), the occurrence of caries in both groups was minimal (varying between 0 % and 4 %). At every time interval, the difference in caries incidence between the two varnish groups was not significant (p > 0.05).

**Table 1:** Demographic details.

Variable	Resin-based FV	Alcohol-based FV	p-value
**Age**	7.48 ± 0.55	7.69 ± 0.47	0.058
**Gender**	Male: 13 (31 %)	Male: 11 (26.2 %)	0.810
	Female: 29 (69 %)	Female: 31 (73.8 %)	

Independent *t*-test; Fisher exact test.

**Table 2:** Comparison of caries incidence between the two groups.

Interval	Resin-based		Alcohol-based		p-value
**Baseline**	0/163	0.0 %	0/167	0.0 %	–
**6 months**	3/159	1.9 %	5/167	3.0 %	0.724
**12 months**	6/149	4.0 %	5/158	3.2 %	0.765
**18 months**	0/131	0.0 %	1/146	0.7 %	1.000
**24 months**	3/131	2.3 %	2/133	1.5 %	0.683

Fisher-exact test; n denotes number of teeth assessed at that interval.

Table 3 contrasts the occurrence of early and advanced caries lesions in resin-based and alcohol-based fluoride varnish groups, using ICDAS-II scoring. The occurrence of early versus advanced caries lesions was evaluated between the fluoride varnish groups using resin-based and alcohol-based formulations at four follow-up intervals: 6, 12, 18, and 24 months. Throughout all follow-up periods (6, 12, 18, and 24 months), the occurrence of advanced caries lesions stayed very minimal in both groups. At no time point was there a statistically significant difference between the resin-based and alcohol-based fluoride varnishes regarding the progression to advanced caries (p > 0.05).

**Table 3:** Comparison of early and advanced caries lesion incidence between the two groups.

Interval	Group	Sound n (%)	Early n (%)	Advanced n (%)	p-value
**6 months**	**Resin**	139 (87.4)	19 (11.9)	1 (0.6)	1.000
	**Alcohol**	147 (88.0)	19 (11.4)	1 (0.6)	
**12 months**	**Resin**	132 (88.6)	17 (11.4)	0	0.428
	**Alcohol**	136 (86.1)	20 (12.7)	2 (1.3)	
**18 months**	**Resin**	118 (90.1)	13 (9.9)	0	0.571
	**Alcohol**	128 (87.7)	18 (12.3)	0	
**24 months**	**Resin**	118 (90.1)	11 (8.4)	2 (1.5)	0.158
	**Alcohol**	115 (86.5)	18 (13.5)	0	

Fisher-exact test; ICDAS-II Score 0 = Sound; 1–2 = Early; 3–6 = Advanced.

**Fig. 2:**
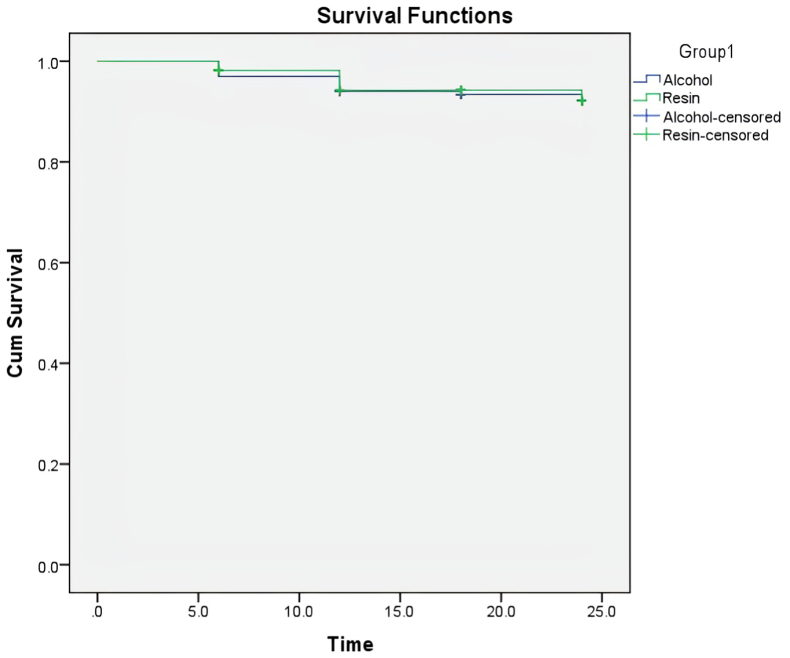
Kaplan–Meier Survival Curves Cumulative caries-free survival rates for resin-based and alcohol-based fluoride varnishes over 24 months, showing comparable effectiveness between groups.

Table 4 outlines the frequency of dental caries and the count of censored instances during the study period for two groups utilizing different types of fluoride varnishes: alcohol-based and resin-based. Caries was evaluated using the ICDAS-II index, with scores 0 and 1 classified as non-carious, while scores 2 and higher were deemed carious. In the alcohol-based varnish group, there were 13 carious occurrences (7.80 %), whereas the resin-based varnish group showed 12 occurrences (7.40 %). The other participants in each group were censored, indicating they either stayed caries-free during the observation period or exited the study (92.2 % in alcohol-based compared to 92.6 % in resin-based). The findings (χ^2^ = 0.002, p = 0.961) show that there was no statistically significant variation in caries occurrence between the two groups, implying similar effectiveness in preventing caries throughout the follow-up duration. Fig. 2 (Kaplan–Meier survival plot) shows the cumulative percentage of participants who stayed caries-free throughout the 24-month observation period in both treatment groups. Both groups showed extremely high survival rates, staying near 1.0 (100 %) for nearly the entire follow-up duration.

**Table 4:** Comparison of caries occurrence among two groups.

Group	N	Event (Caries)	Censored	χ^2^ value	p-value
**Resin-based**	163	12 (7.4 %)	151 (92.6 %)	0.002	0.961
**Alcohol-based**	167	13 (7.8 %)	154 (92.2 %)		
**Overall**	330	25 (7.6 %)	305 (92.4 %)		

Log-rank test.

Table 5 presents the findings from the Intention-to-Treat (ITT) analysis and the per-protocol analysis. Both ITT and Per-Protocol evaluations showed no statistically or clinically meaningful difference in caries scores between the two varnish groups. The effect sizes (Cliff's Delta ≈0.04–0.05) stayed negligible, indicating little practical advantage of Resin-based varnish. The alignment between ITT and Per-Protocol outcomes strengthens the reliability of results and verifies that dropouts did not create attrition bias affecting the outcomes.

**Table 5:** Intention to treat and dropout analysis.

Analysis	Group	Mean ± SD	95 % CI	p-value	Effect Size
**ITT (LOCF)**	**Resin-based FV**	0.25 ± 0.75	0.135–0.365	0.285	0.042
	**Alcohol-based FV**	0.29 ± 0.76	0.175–0.405		
**PP (Completers)**	**Resin-based FV**	0.29 ± 0.80	0.153–0.427	0.302	0.047
	**Alcohol-based FV**	0.34 ± 0.80	0.204–0.476		

ITT = Intention-to-Treat; LOCF = Last Observation Carried Forward; PP = Per-Protocol. n denotes number of teeth analyzed (ITT: Resin = 163, Alcohol = 167; PP: Resin = 131, Alcohol = 133). Mann–Whitney *U* test used due to non-normal distribution. Cliff's Delta interpreted as negligible for both analyses.

The financial cost-benefit analysis revealed a marked difference between the two varnishes. Assuming a restorative cost of ₹2000 per tooth, the alcohol-based varnish achieved ₹62.22 in savings for every ₹1 spent, while the resin-based varnish yielded only ₹8.47. The cost to save ₹100 in future restorative expenses was ₹1.61 for the alcohol-based and ₹11.80 for the resin-based varnish, making the former 7.3 times more economical. Although resin-based varnish showed a slight clinical advantage (0.05 fewer carious lesions per person), its higher cost was unjustified. Thus, alcohol-based varnish offers a more cost-effective and practical preventive option for widespread dental use.

## 4 Discussion

This randomized clinical trial analyze and contrasted the effectiveness and cost-efficiency of two different 5 % sodium fluoride varnish systems—a resin-based product (Profluorid, VOCO) and an alcohol-based option (Fluoritop SR, ICPA)—in preventing dental caries in the first permanent molars of children aged 6–8 years over a duration of 24 months. Since the study population consisted of 6–8-year-old school children with fully erupted and caries-free first permanent molars, caries risk status could not be determined from permanent teeth. Therefore, assessment of caries experience in the primary dentition was used as the basis for risk classification. In line with the American Academy of Pediatric Dentistry (AAPD) guidelines, the presence of one or more carious lesions in the primary dentition was considered indicative of high caries risk. The main results show that both fluoride varnishes had similar clinical effectiveness in caries prevention, revealing no statistically significant differences in caries occurrence, lesion advancement, or survival rates. Importantly, a significant economic difference was noted, with the alcohol-based formulation being considerably more economical. These findings carry significant consequences for public health approaches, especially in settings with limited resources.

The demographic similarity between the two study groups, shown by comparable mean ages (7.48 ± 0.55 years for resin-based and 7.69 ± 0.47 years for alcohol-based) and gender distributions, enhances the internal validity of the comparative results. This guarantees that any noted differences or similarities in caries prevention can be reliably linked to the interventions instead of confusing demographic factors. The consistently low rate of caries noted in both groups during the 24-month follow-up period (cumulative event rates of 7.4 % for the resin-based group and 7.8 % for the alcohol-based group, with a p-value of 0.961) strongly reinforces the recognized protective benefits of fluoride varnish in children's dental care. This corresponds with extensive evidence supporting fluoride's function in enhancing enamel remineralization and preventing demineralization, thus slowing lesion advancement.^1^, ^2^, ^3^, ^4^ Recent research continues to support the overall efficacy of fluoride varnish in preventing tooth decay in children.^16^, ^17^, ^18^, ^19^, ^20^, ^21^ The extended protection noted in this study, with over 90 % of treated teeth staying caries-free for two years as shown by the Kaplan–Meier survival curves (Fig. 2), is due to the elevated fluoride concentration (5 % NaF) in both products. The elevated concentration of fluoride promotes the creation of calcium fluoride-like deposits on enamel surfaces, ensuring a prolonged release of fluoride ions over time, essential for preventing cavities in the long term.^22^^,^^23^

The current results align with earlier systematic reviews and randomized controlled trials that noted no meaningful differences in clinical efficacy between different fluoride varnish preparations.^5^^,^^6^^,^^9^^,^^10^ A recent randomized clinical trial that compared silver diamine fluoride to 5 % sodium fluoride varnish for stopping proximal caries also revealed similar effectiveness between the two fluoride-based substances in treating early lesions.^17^ This strengthens the idea that the particular medium (resin or alcohol) for 5 % sodium fluoride might not greatly change its essential anti-cariogenic characteristics, as long as the fluoride concentration and application methods are sufficient.

Additional examination employing the International Caries Detection and Assessment System II (ICDAS-II) scoring indicated that the occurrence of advanced lesions continued to be minimal during the study, whereas early lesions exhibited only slight, non-statistically significant differences among the groups (p-values varying from 0.158 to 1.000, Table 3). This is an important observation because it indicates that both types of varnish are effective not only in stopping the formation of cavitated lesions but also in managing and possibly reversing early demineralization. This result aligns with the tenets of minimal intervention dentistry, highlighting the importance of early detection and non-invasive approaches to caries management.^5^ The literature has extensively documented fluoride varnish's effectiveness in stopping the advancement of incipient enamel lesions.^24^ The findings of the study are made more reliable through the use of both Intention-to-Treat (ITT) and per-protocol (PP) analyses (Table 5). Both analytical methods resulted in minor differences in average caries scores between the two groups (ITT: 0.25 vs. 0.29; PP: 0.29 vs. 0.34), showing non-significant p-values and small effect sizes (Cliff's Delta ≈0.04–0.05). This consistency effectively eliminates attrition bias caused by participant dropouts and highlights the reliability and internal validity of the results, in accordance with best practices in clinical trial methodology.^6^^,^^10^

Although the clinical effectiveness was similar, a considerable and clinically important difference arose in the economic efficiency of the two varnish systems (Table 6). The alcohol-based varnish showed an impressive cost-to-savings ratio of 1:62.22, greatly exceeding the resin-based variant's ratio of 1:8.47. This means spending only ₹1.61 on the alcohol-based varnish to avoid ₹100 in future restorative treatment expenses, as opposed to ₹11.80 for the resin-based choice. These numbers clearly indicate that even though there may be a slight clinical benefit of resin-based varnish (which was neither statistically nor clinically significant in this research, showing merely 0.05 fewer carious lesions per participant in the ITT analysis), its increased cost is not warranted by its economic benefit, especially in situations where budget efficiency is critical. Recent economic assessments of fluoride varnish initiatives in various contexts also emphasize the significance of cost-effectiveness in public health measures.^18^^,^^19^ However, the economic interpretation of this study is limited by the exclusion of indirect and program-related costs. Expenses such as caregiver time, productivity loss, staff training, administrative overhead, and infrastructure utilization were not included in the analysis. Therefore, the findings represent a financial cost-benefit assessment from a provider and patient expenditure perspective rather than a full societal cost-effectiveness analysis.

**Table 6:** Cost-benefit analysis.

Metric	Resin-based	Alcohol-based
**Cost-to-Savings Ratio**	1:8.47	1:62.22
**Cost per ₹100 saved for patient**	11.80	1.61

Perspective: Societal + patient combined.

These findings are radical from a public health standpoint. The exceptional cost-effectiveness of the alcohol-based varnish, along with its similar clinical efficacy, makes it a practical and sustainable option for extensive caries prevention initiatives, particularly in low- and middle-income nations or underserved populations. The reduced unit price and easier application process lead to less chair time and fewer logistical hurdles, making it perfect for widespread use in school or community programs.^11^^,^^18^^,^^21^ The simplicity of application also allows for task-shifting to support dental staff, which further improves program scalability and accessibility, a feature proven to lower costs in other research.^19^ This corresponds with the worldwide need to deliver affordable and accessible preventive oral healthcare to children, especially in regions with inadequate dental resources. Moreover, the fact that neither product resulted in considerable lesion progression over time underscores their substantial caries-arresting capability. This justifies their role in minimal intervention dentistry approaches, which promote a change from surgical interventions to early identification and non-invasive care for cavities.^5^

This randomized controlled trial featured multiple methodological advantages, including a double-blinded approach, school-based implementation, and a 24-month follow-up, which together boost internal validity and real-world relevance. The application of both Intention-to-Treat and Per-Protocol analyses further enhances the strength of the findings by confirming that attrition did not significantly skew the results. Nevertheless, certain limitations persist. Even though participants were chosen based on a high risk for caries, there was no matching of baseline caries experience, which could introduce variability in susceptibility to caries. The single-site nature of the sample might restrict the generalizability to wider populations with varying demographic or socioeconomic characteristics. While examiner calibration was conducted, minor differences related to the operator during varnish application cannot be entirely ruled out. Additionally, cost estimates were derived from local pricing and treatment costs, which may vary across different healthcare systems. The economic interpretation is limited by the exclusion of indirect and program-related costs; therefore, the analysis does not represent a full societal perspective. Future studies incorporating comprehensive cost domains are required to establish true societal cost-effectiveness. Lastly, the sample size was calculated based on the expectation of a relatively large effect, meaning smaller differences between groups may have gone unnoticed. Future multicenter studies with a wider range of participants, inclusion of patient-reported outcomes, and standardized economic evaluations are suggested to confirm and expand these findings.

## 5 Conclusion

This research offers a robust proof that both resin-based and alcohol-based 5 % sodium fluoride varnishes are similarly effective at preventing dental caries in permanent molars in children throughout a 24-month duration. Nonetheless, the alcohol-based fluoride varnish clearly stands out as the more financially advantageous choice, providing significant savings while maintaining clinical effectiveness. These results hold crucial significance for public oral health policies, especially in areas where economic limitations present a major obstacle to widespread preventive care. Dental professionals, health authorities, and legislators are highly urged to view alcohol-based fluoride varnishes as an effective, efficient, and fair approach for extensive caries prevention efforts in children.

## Patient's/guardian's consent statement

Prior to enrolment in the study, parents, guardians or next of kin provided written informed consent for the minors to participate in this study.

## Ethical clearance statement

Ethical clearance for this study was obtained from the Institutional Ethical Committee of Institute of Medical Sciences, Banaras Hindu University, Varanasi (Approval Number: Dean/2022/EC/3621). The trial was prospectively registered in the Clinical Trials Registry – India (CTRI/2023/06/054321). The study adhered to the CONSORT guidelines (2022) for randomized clinical trials and was conducted in accordance with the Declaration of Helsinki (2013), ensuring the ethical principles and guidelines for conducting human clinical research.

## Funding

This study was financially supported by the Research Grants under Institute of Eminences Scheme, 10.13039/501100002742Banaras Hindu University, sanctioned under order number R/Dev/D/IoE/Equipment/Seed/Grant II/2022-23/4868.

## Declaration of competing interest

The authors declare that they have no known competing financial interests or personal relationships that could have appeared to influence the work reported in this paper.
