# Supplementary material for: Clinical effectiveness and cost-effectiveness of two sodium fluoride varnish systems in preventing dental caries in children: a randomized clinical trial
Source: J Oral Biol Craniofac Res. 2025 Dec 2;16(1):153–9. doi: 10.1016/j.jobcr.2025.11.018 (PMC12719153; doi:10.1016/j.jobcr.2025.11.018)
Supplement: Multimedia component 1 [file mmc1.docx]

**Supplementary Table 1. Input Parameters and Group-Level Economic Outcomes Used for Cost-Benefit Analysis**

| **Parameter / Metric** | **Alcohol-based Fluoride Varnish (Fluoritop Group)** | **Resin-based Fluoride Varnish (Voco Group)** | **Notes** |
| --- | --- | --- | --- |
| **Cost per application (₹)** | 30 | 220 | Expense incurred by dentist |
| **Number of applications per tooth** | 4 | 4 | Every 6 months till 24 months |
| **Total varnish cost per tooth (₹)** | 120 | 880 | Cost/app × 4 |
| **Investment by Dentist (Group Total)** | ₹ 4,950 | ₹ 35,640 | Actual expenditure recorded |
| **Estimated cost of restorative treatment per tooth (₹)** | 2,000 | 2,000 | Assumed standard clinical cost |
| **Total Patient Restoration Cost Saved (Group)** | ₹ 3,08,000 | ₹ 3,02,000 | Based on avoided caries (Table 2 outcomes) |
| **Net Savings (Benefit − Investment)** | ₹ 3,03,050 | ₹ 2,66,360 | From savings minus dentist cost |
| **Return on Investment (ROI)** | 6123% | 747% | [(Net savings / Investment) × 100] |
| **Cost-to-Savings Ratio** | **₹1 : ₹62.22** | **₹1 : ₹8.47** | From Table 6 |
